# Pedestrian Involved Road Traffic Accidents – Case study in Kandy town, Sri Lanka

**Published:** 2019-08

**Authors:** Jayalath Edirisinghe

**Affiliations:** ^*a*^Senior lecturer at University of Peradeniya, Sri Lanka.

## Abstract

**Background:**

It is a known fact that road traffic accidents are responsible for nearly 1.3 million deaths and another 50 million injuries in a year around the globe. Depending on the economic development of the country, victim category may change. In countries like Sri Lanka Vulnerable Road Users (VRU) are more susceptible for road traffic accidents. According to police records pedestrian involved accidents were top the list among other victim categories up to 2015. At present motor cycle riders are recorded as the highest number of victims due to road traffic accidents and pedestrians are at the second place. This paper is about pedestrians involved road traffic accidents reported to Kandy Police station in Sri Lanka to investigate and report a comparison of accidents recorded. Data Collection By law it is mandatory to report all the road traffic accidents to the police station concerned. For this study, road traffic accidents reported to Kandy police station during the period 29.08.2017 to 31.12.2018 were considered. Kandy is the second largest city in Sri Lanka and Kandy has nearly 10% of total traffic related incidents reporting compared to rest of the Country. As explained by Ming Sun et al it is very important pay detailed information about the pedestrian involved accidents to find an acceptable solution to ensure their safety. As a start, summary of accident categories are summarized in the table 1. Table 1: Summary of victim categories reported to Kandy Police Station Severity Gender of the Victim Female Male Grand Total Pedestrian Others Pedestrian Others Fatal 13 2 10 10 35 Grievous 30 19 36 63 148 Grand Total 43 21 46 73 183 According to the table 1, it is evident that pedestrians are highly vulnerable to road traffic accidents in the study area concerned. Further, Table 2 is representation of the same data set in percentages. Table 2 : Percentages of road traffic accidents for Pedestrians and others Pedestrian Others Pedestrian Others Fatal 37.14% 5.71% 28.57% 28.57% 100.00% Grievous 20.27% 12.84% 24.32% 42.57% 100.00% It is important to check whether the accidents are prominent in weekdays or weekends. Table 3 is a summary of accidents summarized according to the day of the week; Table 3 Accident variation according to the day of the week Pedestrian Involved accident Other Accidents Weekdays 70 70 Weekends 19 23 Accident / day (Weekday) 14 14 Accident/ Day (Weekend) 9.5 11.5 According to the dataset considered, there is no difference of accidents per day for weekdays for pedestrian involved and others. But for Weekends, there is a difference of accidents per day on weekends. This may be due to more pedestrians travel during weekdays due to official commitments, schooling etc. Table4. Vehicles involved in Accidents Vehicle Category Victim Category Pedestrian others Motor cycle 31 17 Three wheelers 9 37 Other Vehicles 49 39 Grand Total 89 93 According to the table 4, Motor Cycles and Three wheelers are responsible for most of the Pedestrian involved accidents. Since Motor cycles and three wheelers also categorized as vulnerable road users similar to pedestrians, they should be educated about the importance of sharing facilities with their fellow road users. Table 5 Accident locations Female Male Pedestrian Others Pedestrian Others Fatal Pedestrian crossing 6 0 2 0 others 7 2 8 10 Grievous Pedestrian crossing 9 0 11 1 Others 21 19 25 62 Grand Total 43 21 46 73 as per the information collected and analyzed, pedestrian involved accidents are high at pedestrians crossings compared to other locations. It is a known fact that pedestrian movements, especially across roads are higher at pedestrian crossings, but making such places safer also an important responsibility of the authorities.

**Conclusions:**

Pedestrian involved accidents are at a very high percentage. This may be because of low property damage and minor injury relate accidents also considered. Due to various uncertainties and possible underreporting, only fatal accidents and accidents categories as grievous are considered. According to the dataset considered, pedestrians are at high risk on roads compared to other road users. Main culprits in making pedestrian related accidents are other groups of vulnerable road users namely Motorcycle riders and Three-wheeler drivers. Pedestrian related accidents are more prominent during weekends compared to other victim categories.

**Keywords:**

Traffic Accidents, Pedestrian accidents, Vulnerable road users

